# Lipase-rich fermented solids produced by *wickerhamiella osmotolerans*: direct application as biocatalysts for dairy wastewater treatment and integration with coagulation/flocculation/sedimentation

**DOI:** 10.1007/s42770-026-02145-x

**Published:** 2026-11-13

**Authors:** Jéssyca Ketterine Carvalho, Rosemeire Aparecida Silva-Lucca, Maria Luiza Fernandes Rodrigues, Salah Din Mahmud Hasan, Thais Souto Bignotto, Adriana Fiorini Rosado, Marcia Regina Fagundes-Klen, Edson Antônio da Silva, Jeferson Klein, Marina Kimiko Kadowaki, Cleide Viviane Buzanello, Joel Gustavo Teleken

**Affiliations:** 1 Department of Engineering and Exact Sciences, Federal University of Paraná, Palotina, Paraná Brazil; 2 Center for Engineering and Exact Sciences, State University of Western Paraná, Toledo, Paraná Brazil; 3 Graduate Program in Pharmaceutical Sciences, Center for Medical and Pharmaceutical Sciences, Western Paraná State University (UNIOESTE), Cascavel, Paraná Brazil

**Keywords:** Enzymes, Biocatalysis, Biotechnology, Agro-industrial residues, Coagulation/flocculation/sedimentation

## Abstract

Lipase-based bioprocesses are promising alternatives for the treatment of lipid-rich industrial effluents; however, enzyme recovery and purification steps may limit their practical application. In this study, lipase-rich fermented solids produced by *Wickerhamiella osmotolerans* under solid-state fermentation were evaluated as direct biocatalysts for dairy wastewater treatment. A total of 39 yeast isolates obtained from poultry slaughterhouse wastewater were screened for lipolytic activity. Five isolates with the highest enzymatic indices were selected for molecular identification and identified as Wickerhamiella osmotolerans based on sequencing of the D1/D2 region of the 26 S rDNA. Lipase production was evaluated through sequential experiments investigating particle size, substrate/support composition, moisture content, fermentation time, and inducer addition. Although 100% sunflower seed produced the highest lipolytic activity, the sunflower seed/rice husk mixture (1:1) was selected as a practical trade-off between enzyme production and the incorporation of an agro-industrial residue. Under the selected conditions of 60% initial moisture and 24 h of fermentation, this mixture reached 24.14 U g⁻¹ without external lipid inducers. The direct application of the fermented solid resulted in 65.4% apparent O&G removal but did not reduce turbidity. The subsequent C/F/S step reduced turbidity from 1190 to 693 NTU, corresponding to a stage-specific reduction of 41.8%, but did not provide additional O&G removal. These findings demonstrate a proof-of-concept strategy for simplifying lipase application by eliminating enzyme extraction steps and integrating microbial enzyme production with wastewater treatment.

**Graphical abstract:**

**Figure:**
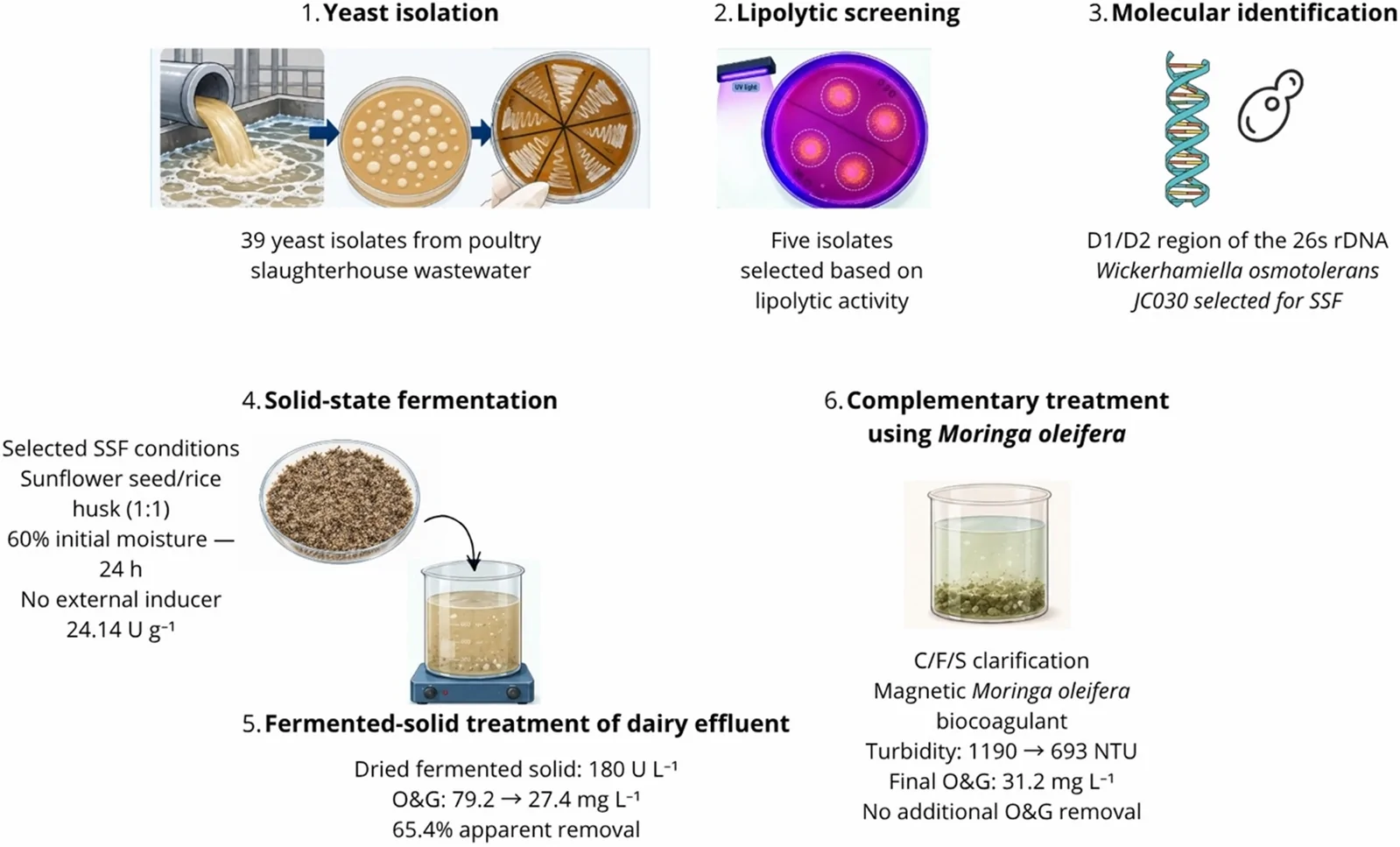

## Introduction

Enzymes are selective biocatalysts that operate under relatively mild conditions and have broad industrial and environmental applications [1–6]. Among them, lipases (triacylglycerol acylhydrolases, EC 3.1.1.3) belong to the group of lipolytic enzymes and catalyze the hydrolysis of triacylglycerols into glycerol, free fatty acids, and partial glycerides [7, 8]. Lipases can be obtained from animal, plant, and microbial sources. Microbial lipases are particularly relevant because bacteria, filamentous fungi, and yeasts can be cultivated using diverse substrates and production systems. Frequently investigated producers include species of *A*s*pergillus*,* P*e*nicillium*,* Rhizopus*, *Candida*, and *Pseudomonas* [8–10].

Microbial lipases can be produced by submerged fermentation (SmF) or solid-state fermentation (SSF). In SmF, microorganisms grow in a liquid nutrient medium containing abundant free water, whereas SSF involves microbial cultivation on a moist solid matrix with little or no free-flowing water [11, 12]. SSF has been investigated for microbial lipase production using lipid-rich substrates and agro-industrial materials [11, 13, 14]. In addition to producing extracellular enzymes, SSF generates fermented solids containing enzymes, residual substrate, microbial biomass, and fermentation metabolites. These materials may be applied directly as biocatalytic matrices, avoiding enzyme extraction and purification in the experimental workflow [15, 16]. Nevertheless, their complexity makes it difficult to attribute treatment effects exclusively to enzymatic activity because adsorption, physical retention, biomass-associated processes, and sedimentation may occur simultaneously.

Bioremediation involves the use of microorganisms, enzymes, or other biological systems to transform contaminants and facilitate their removal from polluted environments [17]. In lipid-rich wastewater, the targeted application of microbial lipases can promote triglyceride hydrolysis, generating glycerol, free fatty acids, and partial glycerides [9, 16, 18]. However, hydrolysis represents lipid transformation and does not, by itself, demonstrate pollutant removal or mineralization. Changes in aqueous-phase O&G may also reflect redistribution among dissolved, emulsified, suspended, and solid-associated fractions. Therefore, treatment performance should be interpreted using both lipid-transformation indicators and physicochemical parameters.

Coagulation/flocculation/sedimentation (C/F/S) is primarily used to destabilize colloidal and suspended material and promote phase separation [19, 20]. Natural coagulants derived from *Moringa oleifera*, including formulations combined with magnetic materials, have been investigated for wastewater clarification [21, 22]. Enzymatic treatment and C/F/S may therefore perform complementary functions: enzymatic treatment can modify the lipid fraction, whereas C/F/S can promote clarification [22]. However, enzymatic hydrolysis may also alter emulsion stability and the subsequent separation behavior of lipid-derived compounds. Evidence concerning the sequential application of these treatments remains limited, particularly when complex fermented solids are used instead of extracted enzymes.

Because lipid-rich industrial wastewaters have been investigated as potential sources of lipase-producing microorganisms [10, 23, 24], poultry slaughterhouse wastewater was selected in the present study as a prospective source of lipolytic yeasts. Although bacteria and filamentous fungi are commonly investigated as lipase producers, comparatively little information is available on non-conventional yeasts such as *Wickerhamiella osmotolerans*, particularly regarding lipase production under SSF and the direct application of the resulting fermented solids as biocatalytic materials for wastewater treatment.

Therefore, this study aimed to isolate and identify lipolytic yeasts from poultry slaughterhouse effluent, evaluate lipase production by *W. osmotolerans* under sequentially selected SSF conditions, and assess the direct application of the resulting fermented solid to dairy wastewater. A subsequent C/F/S step was evaluated to determine its specific contribution to clarification and aqueous-phase O&G removal. The study was designed as a proof of concept for the direct use of fermented solids, while recognizing that the individual enzymatic and non-enzymatic contributions of the fermented matrix could not be isolated.

## Materials and methods

### Materials

Sunflower seeds (*Helianthus annuus*) used as the primary substrate for solid-state fermentation (SSF), rice husk (*Oryza sativa*), and eggshell residues used as structural supports were obtained from local agro-industrial sources in Paraná, Brazil. Extra virgin olive oil (Galo^®^, Brazil; acidity index 0.5%) and sunflower oil were used as lipid inducers during lipase production assays. Yeast extract, peptone, dextrose, bacteriological agar, gum arabic, phenolphthalein, sodium hydroxide, ethanol, acetone, calcium chloride, and all analytical-grade reagents were acquired from commercial suppliers. Rhodamine B and Tween 80 were purchased from Synth (Brazil). Sodium phosphate buffer solutions were prepared using analytical-grade reagents and deionized water. Molecular analyses included reagents for manual DNA extraction, PCR amplification, and electrophoresis, as well as the microsatellite primer (GTG)₅ and the NL1/NL4 primers used for sequence-based identification. Sequencing was performed by Sanger dideoxy sequencing on a MegaBACE™ 1000 automated sequencer. *Moringa oleifera* seeds used for coagulation/flocculation/sedimentation (C/F/S) assays were obtained from local producers. FeSO₄ and Fe(NO₃)₃·9 H₂O were used as precursors for the synthesis of a magnetic Fe₃O₄ material. Poultry slaughterhouse effluent and dairy wastewater samples were collected from agro-industrial facilities located in western Paraná, Brazil.

### Experimental workflow

**Fig. 1:**
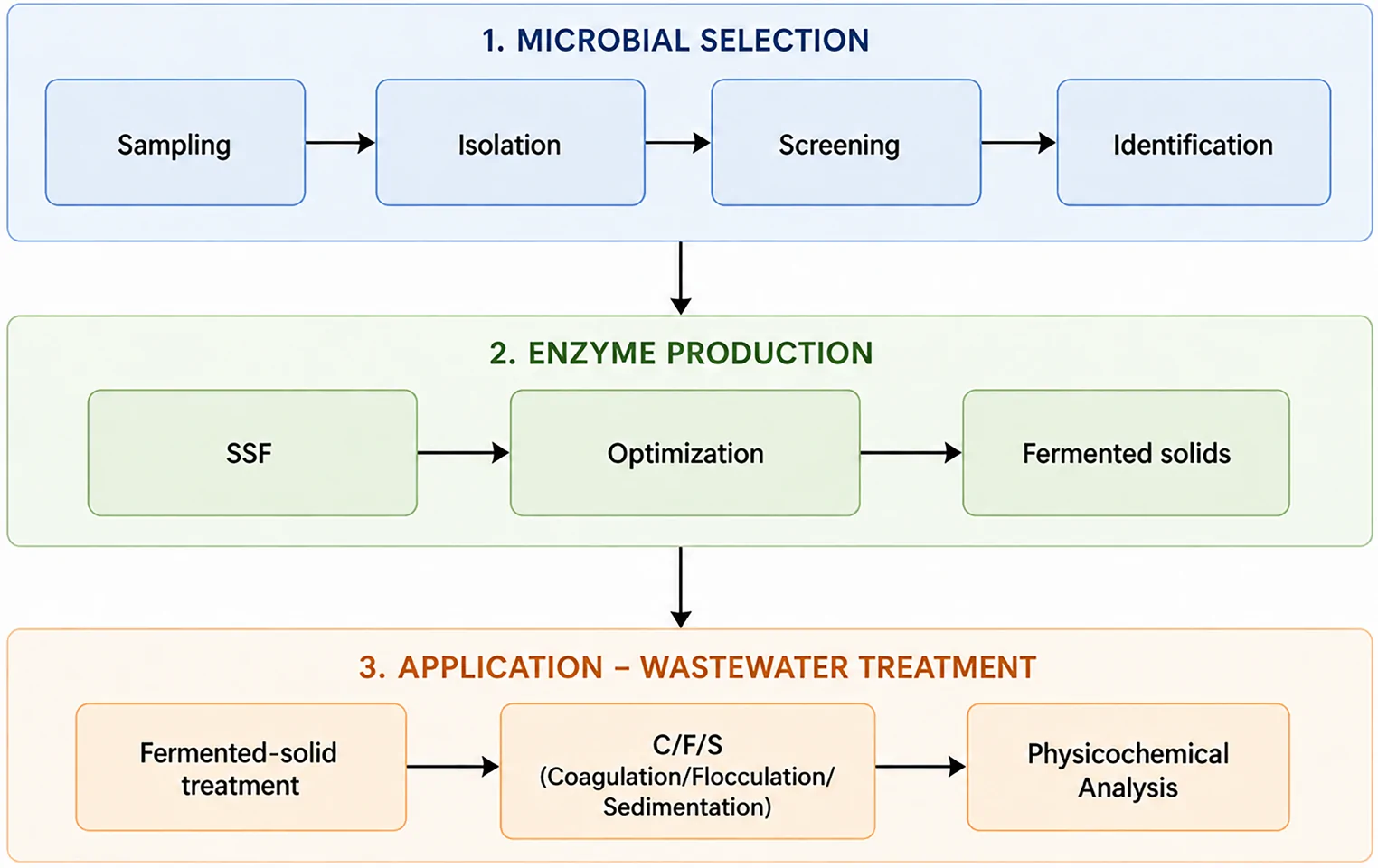
Schematic representation of the experimental workflow, including yeast isolation from lipid-rich effluent, screening and molecular identification of lipase-producing strains, sequential evaluation and selection of SSF conditions for lipase production, and application of the fermented solids in dairy wastewater treatment followed by C/F/S

The experimental design consisted of four sequential stages: (I) isolation and screening of lipase-producing yeasts from a lipid-rich effluent, (II) molecular identification of selected strains, (III) sequential evaluation and selection of SSF conditions for lipase production, and (IV) application of the fermented solids as biocatalysts in dairy wastewater treatment, followed by C/F/S. The overall workflow of the study is illustrated in Fig. 1.

### Origin of yeasts and characterization of raw poultry slaughterhouse wastewater

The yeasts were isolated from a sample of effluent collected at the inlet of the equalization tank during the slaughter period at an industrial poultry slaughterhouse located in the western region of Paraná, Brazil (24°17’5.09"S 53°49’52.74"W). At this stage, the effluent had undergone preliminary screening to remove coarse residues such as skin and solid particles. The oily effluent was selected for microbial isolation because lipid-rich environments are more likely to harbor lipase-producing microorganisms [24]. The physicochemical characterization of the samples, including pH, BOD (biochemical oxygen demand), COD (chemical oxygen demand), and O&G (oils and grease), was performed according to the *Standard Methods for the Examination of Water and Wastewater* [25].

### Yeast isolation and qualitative lipase screening

The use of lipid-rich effluent as a source of microorganisms was based on the selective pressure imposed by high concentrations of oils and fats, which favors the development of lipolytic microbial communities.

Aliquots of 250 µL of undiluted wastewater were spread onto Petri dishes using sterile glass beads. The plates contained YPD medium composed of yeast extract (10 g L⁻¹), dextrose (20 g L⁻¹), peptone (20 g L⁻¹), and bacteriological agar (20 g L⁻¹), supplemented with 0.05% (w/v) tetracycline hydrochloride to inhibit bacterial growth. Yeast isolation was performed using a procedure adapted from Carvalho et al. [1], with modifications in the sample matrix, antibacterial supplementation, and incubation temperature. The plates were incubated at 30 °C for 48 h. After incubation, individual colonies were selected to represent the distinct macroscopic morphologies observed on the isolation plates. The selected colonies were purified by successive streaking on YPD agar, and culture purity was verified by microscopic examination of cell morphology. This procedure resulted in 39 yeast isolates.

To identify lipase-producing yeasts, selective lipase medium consisting of bacteriological agar supplemented with mineral salts (MgSO₄ 0.2 g L⁻¹, NaCl 0.1 g L⁻¹, yeast extract 0.4 g L⁻¹, KH₂PO₄ 0.4 g L⁻¹, K₂HPO₄ 0.1 g L⁻¹ and bacteriological agar 20.0 g L⁻¹) was used. The medium was supplemented with 1% (v/v) extra-virgin olive oil (acidity index of 0.5%) and 0.001% (w/v) Rhodamine B and was evaluated either without Tween 80 or with 0.01% (v/v) Tween 80. The qualitative screening was performed using three independently prepared plates for each medium condition (*n* = 3). Each isolate was point-inoculated onto the agar surface using biomass from a freshly grown YPD culture. Isolates were classified as lipolytic when an orange fluorescent halo was observed around the colony under UV light at 366 nm after incubation at 30 °C for 96 h [13, 26].

The relative lipolytic potential of the isolates was estimated using the enzymatic index (EI), calculated as the ratio between the mean diameter of the fluorescent hydrolysis halo and the mean colony diameter, according to Eq. 1 [27].

1 $$EI = D_h / Dc$$

where Dₕ is the mean diameter of the fluorescent hydrolysis halo (mm) and Dc is the mean colony diameter (mm).

### Molecular identification and phylogenetic analysis

After isolation, the selected yeast cultures were maintained in BHI broth supplemented with glycerol at − 20 °C. Before DNA extraction, the isolates were reactivated on YPD agar at 30 °C for 48 h. Genomic DNA was extracted manually using an SDS–chloroform/isoamyl alcohol protocol. Briefly, yeast biomass was resuspended in 100 µL of lysis buffer containing 50 mmol L⁻¹ Tris, 250 mmol L⁻¹ NaCl, 50 mmol L⁻¹ EDTA, and 0.3% (w/v) SDS at pH 8.0. After incubation at 65 °C for 30 min, DNA was extracted with chloroform/isoamyl alcohol (24:1), precipitated with isopropanol, washed with cold 70% ethanol, dried, and resuspended in 50 µL of TE buffer. DNA concentration was determined using a NanoDrop™ 2000 spectrophotometer.

PCR fingerprinting was performed using the microsatellite primer (GTG)₅ (5′-GTGGTGGTGGTGGTG-3′). Amplification consisted of 40 cycles of denaturation at 95 °C for 30 s, annealing at 50 °C for 1 min, and extension at 72 °C for 1 min. Amplification profiles were evaluated by electrophoresis in 1.5% agarose gels. The five isolates with the highest enzymatic indices (JC030, JC031, JC032, JC033, and JC039) were selected for molecular identification.

The D1/D2 region of the 26 S rDNA was amplified using primers NL1 (5′-GCATATCAATAAGCGGAGGAAAAG-3′) and NL4 (5′-GGTCCGTGTTTCAAGACGG-3′). PCR consisted of an initial denaturation at 95 °C for 2 min, followed by 35 cycles at 95 °C for 15 s, 54 °C for 25 s, and 72 °C for 20 s, with a final extension at 72 °C for 7 min. Amplicons were verified by electrophoresis in 1.0% agarose gels and purified by EDTA/ethanol precipitation.

Purified PCR products were sequenced by the institutional sequencing facility using Sanger dideoxy sequencing on a MegaBACE™ 1000 automated sequencer (Amersham Biosciences), following the facility’s standard protocol. The resulting sequences were compared with sequences available in the NCBI GenBank nucleotide database using BLASTn. The sequences obtained for all five isolates were identical over the 424-bp region deposited in GenBank and showed 99.53% sequence identity and 100% query coverage relative to the reference sequence of Wickerhamiella osmotolerans strain DMKU VGT1-14 (MN194615), supporting their identification as W. osmotolerans. The sequences obtained for JC030, JC031, JC032, JC033, and JC039 were deposited in GenBank under accession numbers OR608348, OR608349, OR608350, OR608351, and OR608352, respectively.

Phylogenetic analysis was performed using MEGA11. Sequence alignment was performed using the ClustalW algorithm. The phylogenetic tree was constructed using the Neighbor-Joining method [28] and the Kimura two-parameter substitution model [29]. Branch support was evaluated using 1,000 bootstrap replicates. Fusarium phyllophilum AB587006 was used as the outgroup.

### Solid-state fermentation for lipase production

#### Substrate and support characterization

Moisture content was determined gravimetrically using 1.0 g of each homogenized material. Samples were dried in an oven at 105 °C for 24 h, and moisture content was calculated on a wet basis as the mass lost during drying relative to the initial sample mass. Ash content was determined using 3.0 g of homogenized material, which was incinerated in a muffle furnace at 550 °C for 5 h. Ash content was calculated as the mass of the remaining inorganic residue relative to the initial sample mass. Moisture and ash contents were determined according to AOAC procedures [30]. O&G content was determined separately according to Method 5520D of Standard Methods [25]. Three analytical determinations were performed for each material and method.

The selection of sunflower seed as substrate was based on its high lipid content, which acts as an inducer for lipase synthesis, while agro-industrial residues such as rice husk and eggshell were evaluated as structural supports to improve aeration and moisture distribution.

#### Inoculum preparation

The yeast strains JC030, JC031, JC032, JC033, and JC039 were reactivated on YPD agar at 30 °C for 48 h. Biomass from a freshly grown colony of each isolate was aseptically transferred with a sterile inoculating loop to a 250 mL Erlenmeyer flask containing 100 mL of YPD broth supplemented with 0.05% (w/v) tetracycline hydrochloride (0.5 g L⁻¹) to minimize bacterial contamination during inoculum propagation. The cultures were incubated at 30 °C for 48 h. After incubation, the cell concentration in the YPD culture was determined using a Neubauer counting chamber. Based on the measured cell concentration, the required volume of the broth culture was calculated and transferred directly to the SSF substrate, without prior centrifugation or washing, to provide an initial inoculum concentration of 1 × 10⁸ cells g⁻¹ of substrate. Equations (2) and (3):

2 $$\:Cells\:{mL}^{-1}=\:n/5.{10}^{4}.f$$

where:

*n* = Average number of cells in the quadrants of the Neubauer chamber.

5 × 10^4^ = Neubauer chamber constant.

*f* = Dilution factor.

3 $$\:{V}_{ino}=\frac{{G}_{sub}.{G}_{target}}{{C}_{broth}\:{mL}^{-1}}$$

where:

*V*_*i*no_ = Volume of inoculum (mL) to be added per gram of substrate.

*G*_sub_ = Grams of substrate used.

*C*_Target_ = Target initial cell concentration (1 × 10⁸ cells g⁻¹^)^.

C_broth_= cell concentration measured in the inoculum broth (cells mL⁻¹).

The SSF experiments were conducted sequentially. SS was used as the lipid-rich substrate, while RH and ES were evaluated as structural supports. Among the five identified isolates, *W. osmotolerans* JC030 was selected for the SSF experiments based on its lipolytic performance. The experimental sequence comprised evaluation of SS particle size, comparison of 100% SS with the SS/RH and SS/ES mixtures, evaluation of initial moisture content, and assessment of external inducer addition. The conditions selected from these experiments were subsequently used to produce the fermented solid applied to dairy wastewater.

#### General SSF conditions

SSF assays were conducted using 10 g of substrate or substrate/support mixture placed in 150 mL Erlenmeyer flasks. Before moisture adjustment and inoculation, the substrate or substrate/support mixture was autoclaved at 121 °C for 20 min and allowed to cool. Moisture content was then adjusted using sterile 50 mmol L⁻¹ sodium phosphate buffer (pH 7.0), and the inoculum was added to provide an initial concentration of 1 × 10⁸ cells g⁻¹ of substrate. The kinetic experiments used to evaluate the isolates, particle size, and substrate/support composition were conducted at 30 °C for up to 144 h, with sampling at 24-h intervals. Separate fermentation flasks were prepared for each sampling time. At each 24-h interval, three independent flasks were removed from incubation and destructively harvested; therefore, repeated samples were not collected from the same flask. The harvested fermented material was stored at − 20 °C to interrupt fermentation until subsequent processing.

Subsequent experiments evaluating moisture and inducer addition, as well as production of the fermented solid applied to the dairy wastewater, were conducted for 24 h using the conditions selected in the preceding experiments. An uninoculated SSF control was prepared for each substrate or substrate/support system. The uninoculated controls were prepared using the same sterilized substrate mixtures, which were adjusted to the corresponding moisture contents with sterile phosphate buffer, and incubated under the same temperature and time conditions as the inoculated treatments, but without the addition of yeast cells. The uninoculated controls were subsequently dried and processed in the same manner as the fermented samples. After fermentation, the fermented solids were dried in a forced-air oven at 30 °C for 48 h. Lipolytic activity was determined after drying; therefore, all reported activity values correspond to the activity retained in the dried fermented solids.

#### Effect of particle size

**Fig. 2:**
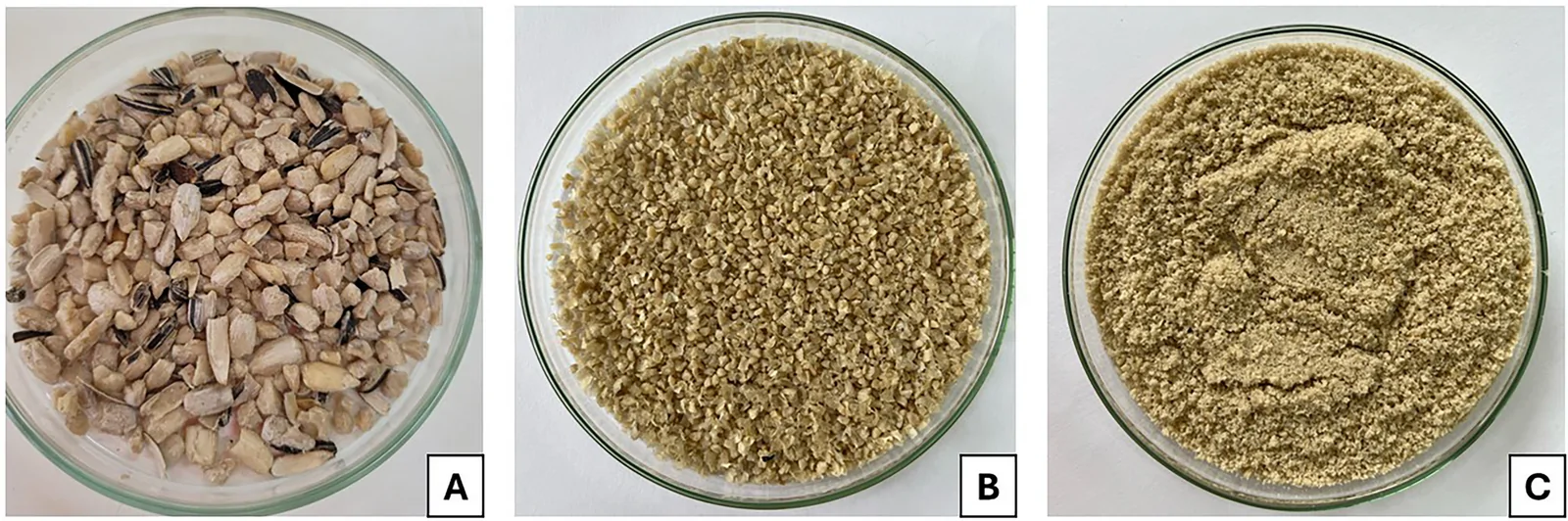
Particle sizes of the SS substrate. The following particle sizes were used: (**A**) 4.75 mm; (**B**) 2.00 mm; (**C**) 1.18 mm

To evaluate the effect of sunflower seed (SS) particle size on lipase production, the methodology proposed by [16] and [22] was followed. The SS were previously ground and then classified into different granulometries using standardized sieves. The particle sizes used were: 4.75 mm (4 mesh), 2.00 mm (10 mesh), and 1.18 mm (14 mesh) (Fig. 2).

#### Effect of support addition

**Fig. 3:**
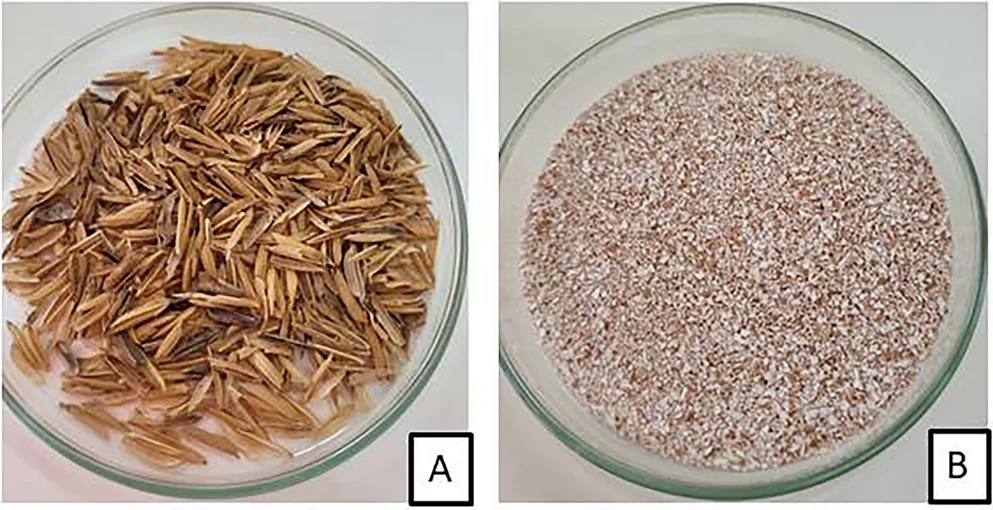
Rice husk (**A**) and eggshell (**B**) supports used in SSF

Two supports classified as agro-industrial residues were used: rice husk (RH) (*Oryza sativa*) and eggshell (ES) (Fig. 3), along with the yeast *W. osmotolerans* JC030, identified as the best lipase producer. The supports were crushed, sieved, and separated into different particle sizes. The combinations were prepared using a mix of SS (1.18 mm) with RH (4.75 mm) and SS (1.18 mm) with ES (1.18 mm). The substrate/support combinations were compared with 100% SS to evaluate the effect of replacing 50% of the sunflower seed with rice husk or eggshell on lipase production.

#### Experimental design and statistical analysis

To investigate the factors affecting lipase production under SSF, substrate composition, initial moisture content, and inducer addition were evaluated in sequential experiments. All fermentation and wastewater-treatment experiments were performed in triplicate using independently prepared experimental units. When repeated measurements were obtained from the same sample, they were treated as analytical replicates and averaged before statistical analysis.

Results were expressed as mean ± standard deviation of three independent experimental replicates. The effects of substrate composition and initial moisture content on lipolytic activity were evaluated by two-way ANOVA, including their interaction. The substrate–inducer experiment was also analyzed by two-way ANOVA, with substrate composition and inducer condition treated as categorical factors. Wastewater-treatment stages were analyzed by one-way repeated-measures ANOVA, with treatment stage as the within-unit factor and each independent experimental unit treated as the subject.

When a significant stage effect was detected in the wastewater-treatment analysis, pairwise comparisons were performed using the within-unit error term with Holm adjustment for multiple comparisons. For the factorial SSF experiments, Tukey’s test was applied to significant effects involving factors with more than two levels or significant interactions. Statistical significance was established at *p* < 0.05. Statistical analyses were performed using TIBCO Statistica, version 14, and R software (R Core Team). Graphs were prepared using Origin, version 8.5.

#### Evaluation of the effect of moisture on SSF for lipase production

The effects of substrate composition and initial moisture content on lipolytic activity were evaluated using a 3 × 3 factorial design. The factors were substrate composition, with three levels (SS, SS/RH, and SS/ES), and initial moisture content on a wet basis, with three levels (50%, 55%, and 60%). Three independent fermentation units were evaluated for each factor combination, resulting in 27 experimental units. Lipolytic activity was analyzed by two-way ANOVA, including the main effects of substrate composition and moisture and their interaction. When significant differences were detected, Tukey’s test was applied at a significance level of *p* < 0.05.

#### Evaluation of the effect of support type combined with inducer use in SSF

The effects of substrate composition (SS/RH and SS/ES) and inducer condition (olive oil, sunflower oil, and no inducer) on lipolytic activity were evaluated using a 2 × 3 factorial design. Both factors were treated as categorical variables. The 2 × 3 factorial design comprised 18 independent experimental runs conducted in random order. The data were analyzed by two-way ANOVA, including the main effects of substrate and inducer and their interaction. Tukey’s test was planned for factors with more than two levels when a significant effect was detected. Statistical significance was established at *p* < 0.05 (Table 1).

**Table 1:** Categorical factors and treatment levels evaluated in the 2 × 3 factorial design

Factor	Categorical levels
Substrate composition	SS/RH; SS/ES
Inducer condition	Olive oil; no inducer; sunflower oil

Legend: SS/RH – sunflower seed/rice husk; SS/ES – sunflower seed/eggshell; OO – olive oil; SO – sunflower oil; N/I – no inducer.

For the wastewater treatment assays, physicochemical parameters including pH, turbidity, O&G, and FFA were expressed as mean ± standard deviation of three independent treatment units. Stage-wise changes in O&G concentration and net apparent removal efficiencies were calculated descriptively from the corresponding mean O&G concentrations. These derived values were not subjected to inferential statistical analysis. Differences among the raw effluent (RE), fermented-solid-treated effluent (FSE), and FSE subsequently subjected to C/F/S were evaluated by one-way repeated-measures ANOVA. Experimental unit was treated as the subject because the three stages were sequentially measured within the same independent units. Pairwise comparisons were adjusted using the Holm method.

### Analytical methods

#### Lipase activity assay

The determination of LA was based on a titration method using NaOH to quantify the free fatty acids released by the enzyme from triacylglycerols [31]. The reaction medium was prepared as an emulsion containing 10% (w/v) powdered gum arabic and 7.15% (w/v) extra-virgin olive oil in 50 mmol L⁻¹ sodium phosphate buffer (pH 7.0). The olive oil, with an acidity index of 0.5%, was used as the reaction substrate. The fermented-solid samples used in the lipolytic activity assay had previously been dried at 30 °C for 48 h. Thus, the measured activity represents the residual activity after the drying procedure.

The samples were placed in 125 mL Erlenmeyer flasks containing 1 g of fermented solid and 20 mL of the emulsion to allow the release of fatty acids. The flasks were incubated in an orbital shaker at 150 rpm for 20 min at 37 °C. After this period, the reaction was stopped by adding 20 mL of an ethanol/acetone solution (1:1 v/v). The released fatty acids were titrated with 50 mmol L⁻¹ NaOH solution containing phenolphthalein as a pH indicator, using a digital burette (Kasv Electronic Burette), until a final pH of 10 was reached. For each substrate or substrate/support system, the corresponding uninoculated SSF control was subjected to the same drying, reaction, and titration procedures. The NaOH volume consumed by this control was used as the matrix-blank volume $\:{(V}_{b})$in Eq. 4 and was subtracted from the mean volume consumed by the corresponding fermented sample.

One unit (U) of LA was defined as the amount of enzyme that released 1 µmol of fatty acids per minute under the assay conditions, calculated using Eq. 4.

4 $$\text{LA } (\text{U g}^{-1}) = \frac{[(V_m - V_b) \times C_{\mathrm{NaOH}} \times f_c]}{(t \times m)}$$

Where:

Vm is the mean NaOH volume consumed in the titration of the fermented sample (mL); Vb is the NaOH volume consumed by the corresponding uninoculated matrix control (mL); CNaOH is the NaOH concentration (mmol L⁻¹); fc is the correction factor of the standardized NaOH solution; t is the reaction time (min); and m is the mass of fermented solid used in the reaction (g).

LA was expressed as enzymatic activity units per gram of fermented solid (U g⁻¹). Therefore, all reported lipolytic activities were corrected for the background acidity of the corresponding uninoculated substrate or substrate/support matrix. LA was determined through three analytical titrations of the fermented material from each independent fermentation flask. The analytical measurements were averaged, and the mean obtained for each flask was used as one independent observation in the statistical analysis.

#### Determination of free fatty acids (FFA)

The free fatty acid (FFA) content of the wastewater samples was determined by titration based on AOCS Official Method Ca 5a-40. A 1.0 mL aliquot of liquid wastewater was mixed with 25 mL of a neutralized ethanol–diethyl ether solution and titrated with standardized 0.01 mol L⁻¹ NaOH using phenolphthalein as the endpoint indicator. FFA content was expressed as oleic acid equivalents on a volume basis (% w/v) and calculated according to Eq. (5):

5 $$\begin{array}{c}\:\mathrm{FFA}\:(\mathrm{\%}\:\mathrm{w}/\mathrm{v}\:\mathrm{as}\:\mathrm{oleic}\:\mathrm{acid})\:\\=\:(\mathrm{VNaOH}\:\times\:\:\mathrm{CNaOH}\:\times\:\:28.2)\\/\mathrm{Vsample}\end{array}$$

where VNaOH is the volume of NaOH consumed in the titration (mL), CNaOH is the molar concentration of the NaOH solution (mol L⁻¹), and Vsample is the wastewater sample volume (mL).

### Dairy wastewater treatment

#### Wastewater characterization

The dairy wastewater was collected at the end of the production process, before any industrial treatment, from a dairy facility located in western Paraná, Brazil (24°46′53.46″S, 53°42′15.10″W). The wastewater was used on the day of collection and maintained at 4 °C between collection and experimental use. Before treatment, the sample was homogenized and divided among three independent experimental units.

The physicochemical parameters were determined separately for each independent experimental unit according to the Standard Methods for the Examination of Water and Wastewater [25]. pH was determined using the electrometric method (Method 4500-H⁺ B), and turbidity was measured using the nephelometric method (Method 2130 B). O&G was determined using Method 5520 B. FFA was determined using the titrimetric procedure described in the preceding subsection. These analyses were performed for the raw effluent (RE), the effluent treated with the lipase-rich fermented solid (FSE), and the effluent obtained after C/F/S.

#### Treatment with lipase-rich fermented solids

The wastewater-treatment assays were conducted in three independent experimental units, each containing 1.0 L of dairy wastewater. The dried fermented solid had a post-drying lipolytic activity of 24.14 U g⁻¹. Therefore, 7.46 g of fermented solid were added to each experimental unit, providing a total lipolytic activity of 180 U L⁻¹. The assays were conducted at 37 °C and 150 rpm for 168 h, following the treatment condition reported by De Moura-Dickel et al. [16].

Although uninoculated controls were used during SSF to correct the lipolytic activity measurements, the corresponding unfermented solid was not applied as a control in the wastewater-treatment assays. Consequently, the experimental design did not allow the enzymatic contribution to be separated from the non-enzymatic effects of the fermented matrix.

The effects of the fermented-solid treatment were evaluated by comparing physicochemical parameters before and after treatment, with emphasis on changes in aqueous-phase oil and grease (O&G) concentration and free fatty acid (FFA) content.

#### Coagulation/flocculation/sedimentation treatment (C/F/S)

Following treatment with the lipase-rich fermented solid, the effluent was subjected to coagulation/flocculation/sedimentation (C/F/S). This complementary treatment was selected because *M. oleifera* seeds are recognized as natural coagulants for turbidity reduction, whereas magnetic Fe₃O₄-based materials may facilitate coagulant separation and recovery [32]. For preparation of the magnetic biocoagulant, *M. oleifera* seed extract was combined with the synthesized magnetic material.

A magnetic Fe₃O₄ material was prepared by aqueous coprecipitation using a procedure adapted from previous studies [33, 34]. Initially, 2.8 g of FeSO₄ and 1.1 g of Fe(NO₃)₃·9 H₂O were dissolved in 10 mL of distilled water. Subsequently, 1.2 g of NaOH was added under continuous mixing, promoting the formation of a dark magnetic precipitate. The precipitate was separated, washed with distilled water, and dried at 50 °C. Because no direct structural, morphological, crystallite-size, or particle-size characterization was performed, the synthesized product is referred to throughout this study as a magnetic Fe₃O₄ material rather than as nanoparticles. Accordingly, nanometric dimensions reported for materials prepared in previous studies were not attributed to the material obtained here.

The biocoagulant solution was prepared using 5 g of dehulled *M. oleifera* seeds, ground with 100 mL of 0.4 mol L⁻¹ CaCl₂ saline solution for 2 min. The mixture was then subjected to magnetic stirring for 30 min and vacuum-filtered using qualitative filter paper, yielding the *M. oleifera* biocoagulant [21].

To prepare the magnetic biocoagulant, 300 mg of the synthesized magnetic material was combined with 100 mL of the M. oleifera biocoagulant. The mixture was sonicated for 5 min and magnetically stirred at 50 rpm for 1 h, following the procedure described by Mateus et al. [33] and Antunes et al. [35]. The resulting magnetic biocoagulant stock suspension was prepared from a *M. oleifera* seed extract corresponding to an initial seed concentration of 50 g L⁻¹ and contained 3.0 g L⁻¹ of the synthesized magnetic material. No direct structural, morphological, or particle-size characterization was performed for the material synthesized in the present study. Therefore, crystallite sizes reported in previous studies using related procedures were not assigned to the material used here.

Because the synthesized magnetic material was not directly characterized by XRD, FTIR, electron microscopy, DLS, or related techniques, no relationship between its structural properties and treatment performance can be established. The results should therefore be interpreted exclusively in terms of the observed C/F/S performance under the evaluated operational conditions.

The FSE was subjected to C/F/S using the magnetic biocoagulant stock suspension prepared with 0.4 mol L⁻¹ CaCl₂. For each test, 7.5 mL of the stock suspension was added to 250 mL of pre-treated effluent, corresponding to a biocoagulant dose of 1,500 mg L⁻¹ expressed as the initial *M. oleifera* seed-mass equivalent. The corresponding dose of magnetic material was 90 mg L⁻¹. For each test, 250 mL of the pre-treated effluent was used. The procedure consisted of an initial rapid mixing step for coagulation, carried out at 100 rpm for 2 min, followed by a slow mixing step for flocculation at 20 rpm for 10 min [21].

Subsequently, the samples were exposed to a magnetic field of 400 kA m⁻¹ for 20 min, promoting sedimentation. The C/F/S process aimed to reduce turbidity in the FSE and assess how post-treatment by C/F/S affected remaining physicochemical parameters.

## Results and discussion

### Characterization of poultry slaughterhouse wastewater used for yeast isolation

The physicochemical characterization of the poultry slaughterhouse effluent used as the source for yeast isolation is presented in Table 2. The evaluated parameters included pH, BOD, COD, and O&G, providing an overview of the organic load and environmental conditions of the wastewater.

**Table 2:** Physicochemical characterization of poultry slaughterhouse wastewater used for yeast isolation

Parameter	Value	Unit
pH	6.09	-
BOD	3,032	mg O₂ L⁻¹
COD	4,009	mg O₂ L⁻¹
O&G	1,275	mg L⁻¹

The physicochemical characterization of the poultry slaughterhouse effluent (Table 2) indicates a wastewater matrix with high organic and lipid loads, making it a relevant source for the isolation of potentially lipolytic microorganisms. The wastewater had a pH of 6.09, which is mildly acidic rather than neutral.

The measured pH represents a mildly acidic condition compatible with the growth of *Saccharomyces cerevisiae*, whereas pronounced growth inhibition has been reported under alkaline conditions at pH 8.0–9.0 [36]. Although pH responses are species-dependent, a pH of 6.09 was therefore unlikely to preclude the recovery of yeast isolates from the wastewater. However, wastewater pH alone does not demonstrate selective enrichment or lipolytic activity, which was subsequently evaluated by culture-based screening. The presence of biodegradable organic matter, as indicated by the BOD and COD values, may support the development of metabolically active microbial communities.

The wastewater contained 3,032 mg L⁻¹ of BOD and 4,009 mg L⁻¹ of COD. BOD represents the oxygen consumed through the microbial degradation of biodegradable organic matter under the conditions of the assay, whereas COD estimates the oxygen equivalent required to chemically oxidize organic compounds and other oxidizable substances. The resulting BOD/COD ratio was 0.76, indicating that a substantial fraction of the oxidizable load was biodegradable. Although the measured concentrations confirm a high organic load, values in slaughterhouse wastewater vary considerably according to water consumption, production scale, processing operations, and the segregation or recovery of blood and solid residues.

More importantly, the presence of O&G represents a key selective pressure driving the enrichment of lipolytic microorganisms. Lipid-rich environments are known to stimulate the expression of lipase-related metabolic pathways, favoring microorganisms capable of utilizing triglycerides as carbon sources [23]. In this sense, the effluent used in this study can be considered not only a contamination matrix but also a selective ecological niche for biocatalyst discovery.

High organic loads have also been reported in poultry slaughterhouse wastewaters [37, 38]. Together with the oily characteristics observed in the sample evaluated in the present study, this supported its selection as a prospective source of lipolytic microorganisms.

### Lipolytic screening, molecular identification, and phylogenetic analysis

**Fig. 4:**
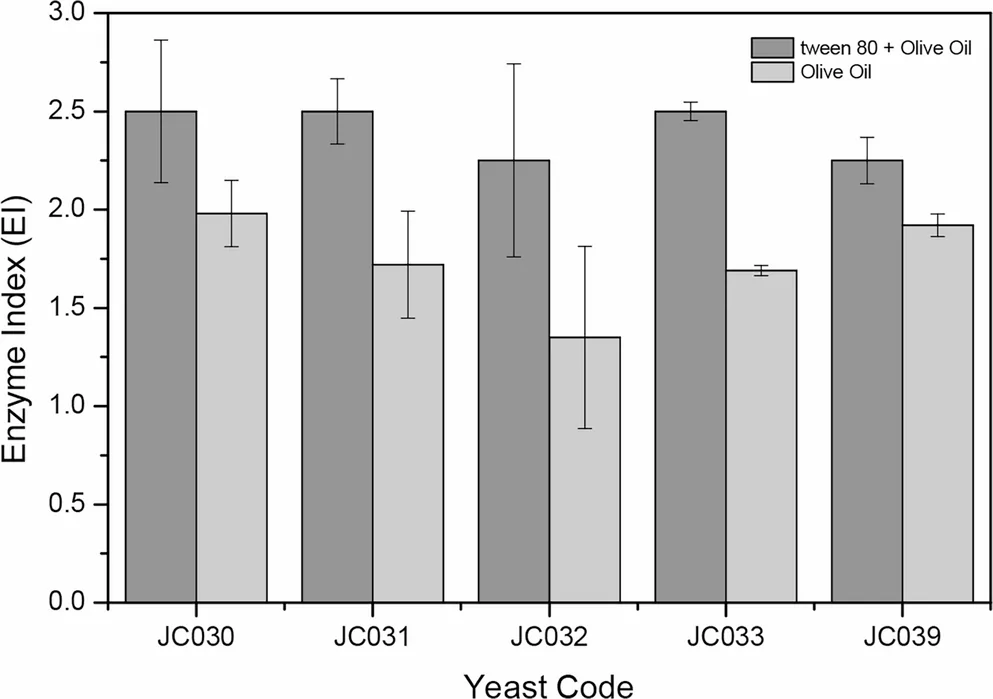
Enzymatic index (EI) of the five selected yeast isolates grown on Rhodamine B agar containing olive oil, either with or without Tween 80. EI was calculated as the ratio between the fluorescent halo diameter and the colony diameter. Bars represent mean values, and error bars represent standard deviations from three independent plates (*n* = 3)

**Fig. 5:**
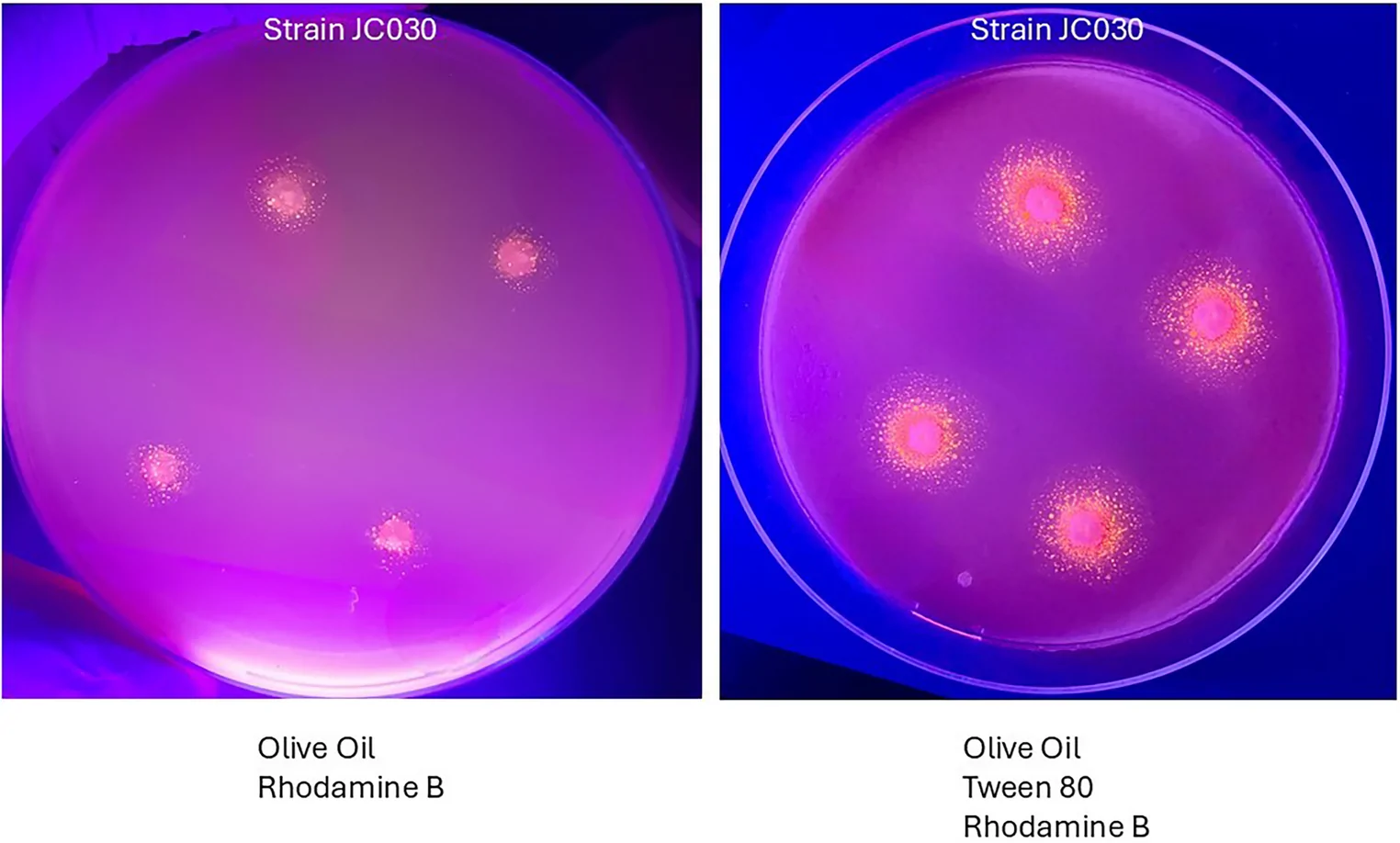
Representative Rhodamine B agar plates showing the lipolytic activity of yeast isolate JC030 after incubation at 30 °C for 96 h and visualization under UV light at 366 nm: (**A**) medium containing olive oil without Tween 80; and (**B**) medium containing olive oil and Tween 80. Orange fluorescent halos around the colonies indicate extracellular lipolytic activity

A total of 39 yeast isolates obtained from poultry slaughterhouse wastewater were subjected to qualitative lipolytic screening on Rhodamine B agar containing olive oil, with or without Tween 80. Isolates were classified as positive when an orange fluorescent halo was observed around the colony under UV light at 366 nm after 96 h of incubation at 30 °C. Twelve of the 39 isolates (30.8%) produced fluorescent halos and were therefore classified as lipolytic in the qualitative screening. The EI values of the five isolates subsequently selected for molecular identification are shown in Fig. 4, whereas representative fluorescent-halo responses are shown in Fig. 5.

Rhodamine B agar containing lipid substrates is widely used for the qualitative detection of lipase-producing microorganisms [9, 39–41]. The fluorescent halo results from the interaction of Rhodamine B with fatty acids released during lipid hydrolysis and was therefore interpreted as evidence of extracellular lipolytic activity. Tween 80 was included as an emulsifying agent to improve olive oil dispersion in the culture medium. However, the classification of isolates as lipolytic was based on fluorescent halo formation rather than on a quantitative comparison between media with and without Tween 80 (Fig. 5).

The isolates classified as positive in the qualitative screening were subsequently compared using the EI, calculated as the ratio between the fluorescent halo diameter and the colony diameter (Fig. 4). Based on the highest EI values, five isolates (JC030, JC031, JC032, JC033, and JC039) were selected for molecular identification (Table 3). All five isolates were identified as *W. osmotolerans*. JC030, JC031, and JC033 showed an EI of 2.50, whereas JC032 and JC039 showed EI values of 2.25. Reports of lipase production by *W. osmotolerans* remain limited, particularly under solid-state fermentation conditions and for environmental applications, indicating that this species is an underexplored source of lipolytic biocatalysts.

**Table 3:** Molecular identification of selected yeast strains with the highest enzymatic indices

Yeast code	Species	Enzyme index
JC030	*Wickerhamiella osmotolerans*	2.50
JC031	*Wickerhamiella osmotolerans*	2.50
JC032	*Wickerhamiella osmotolerans*	2.25
JC033	*Wickerhamiella osmotolerans*	2.50
JC039	*Wickerhamiella osmotolerans*	2.25

**Fig. 6:**
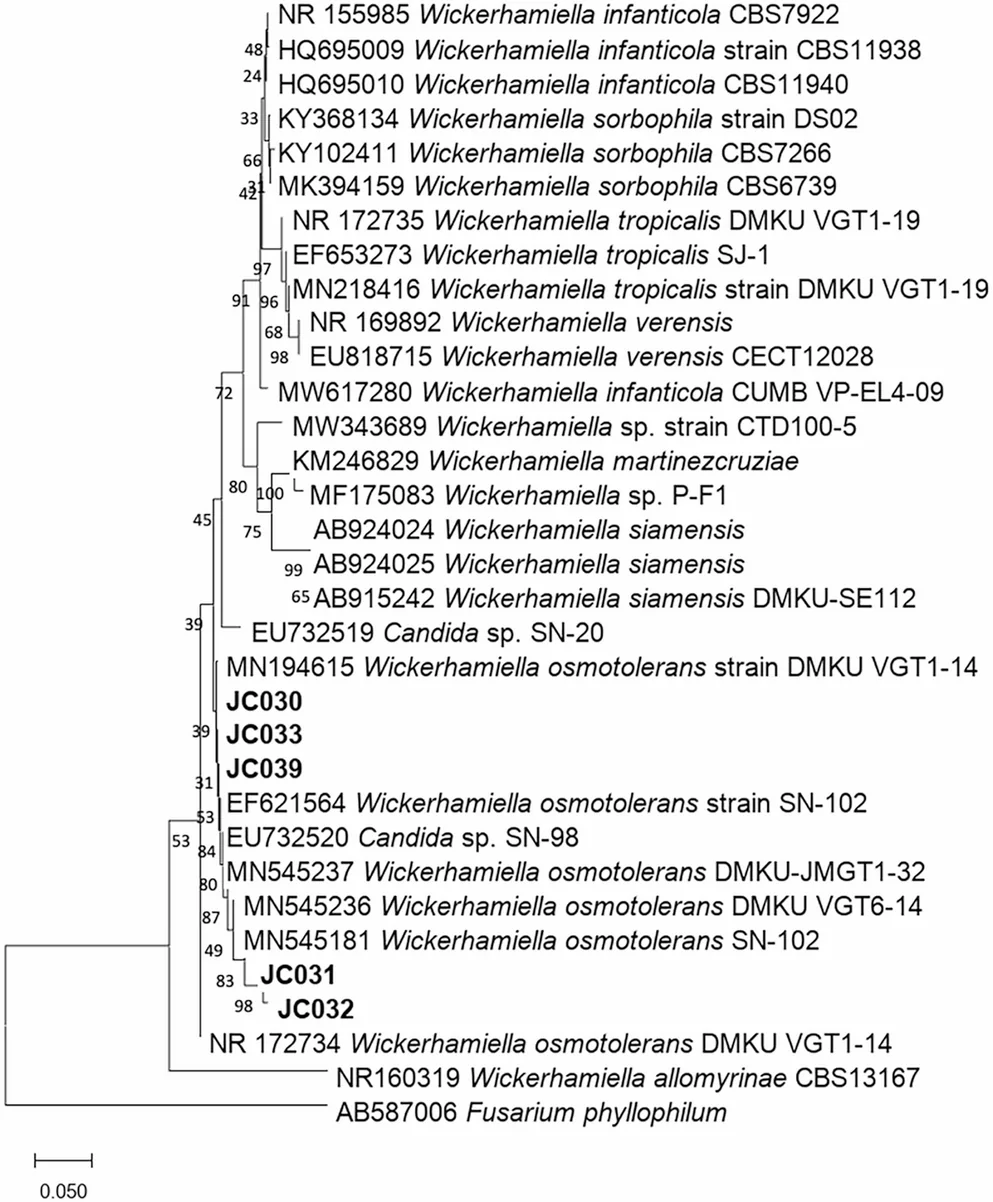
Neighbor-Joining phylogenetic tree of the five selected yeast isolates and related reference sequences. Evolutionary distances were calculated using the Kimura two-parameter model, and branch support was evaluated using 1,000 bootstrap replicates. Bootstrap support values (%) are shown at the nodes. *Fusarium phyllophilum* AB587006 was used as the outgroup. The isolates obtained in this study are shown in bold. Their GenBank accession numbers are OR608348 (JC030), OR608349 (JC031), OR608350 (JC032), OR608351 (JC033), and OR608352 (JC039). The scale bar represents 0.05 nucleotide substitutions per site

Phylogenetic analysis placed the five selected isolates within the *W. osmotolerans* group together with reference sequences, supporting the BLASTn-based identification (Fig. 6). The sequences deposited for the five isolates were identical over the analyzed 424-bp region. Therefore, the marker used in this study did not reveal sequence variation among JC030, JC031, JC032, JC033, and JC039. Moreover, because some internal branches showed low bootstrap support, the phylogenetic tree was used to support taxonomic placement rather than to infer relationships among the study isolates or explain differences in their lipolytic activities.

From a biotechnological perspective, the identification of lipase-producing yeasts from industrial effluents expands the diversity of microbial resources potentially applicable to enzyme production and environmental remediation. The selection of *W. osmotolerans* JC030 for subsequent experiments was therefore based not only on its higher enzymatic index, but also on the potential relevance of investigating a still poorly explored yeast species in SSF-based bioprocesses.

### Solid-state fermentation for lipase production

#### Substrate and support characterization

The physicochemical characterization of the substrates and supports (Table 4) revealed substantial differences in their nutritional and structural properties, which are directly associated with microbial growth and enzyme production under solid-state fermentation conditions.

**Table 4:** Proximate composition analysis of SS, RH, and ES

Physicochemical Composition	SS	RH	ES
Moisture (%wet basis)	4.25 ± 0.19	9.03 ± 0.26	0.41 ± 0.11
Ash (%)	2.86 ± 0.03	13.79 ± 0.70	95.53 ± 0.04
Oil content (%)	49.92 ± 1.44	0.47 ± 0.12	0.09 ± 0.01

Sunflower seed (SS) exhibited the highest oil content (49.92%), a value within the range reported for sunflower genotypes [42]. This high lipid content supports its use as a substrate for lipase production because oils can act as inducers of microbial lipase synthesis [43]. Therefore, SS provided a lipid-rich fermentation matrix potentially favorable for extracellular lipase production.

In contrast, RH and ES presented low oil contents, indicating that their contribution to the fermentation matrix cannot be attributed to lipid supply. The physical characteristics of SSF matrices can influence oxygen transfer, moisture distribution, heat transfer, and substrate accessibility. In a previous mixed-substrate SSF study, RH was considered to provide surface area for fungal growth [44]. Accordingly, RH was evaluated here primarily as a structural component, although its possible nutritional contribution was not separately quantified.

The high ash content observed for ES (95.53%) is consistent with the predominantly mineral composition of eggshell, which consists mainly of calcium carbonate [45]. However, matrix pH, porosity, and microbial colonization were not measured; therefore, the effect of eggshell composition on fermentation performance cannot be assigned to a specific mechanism.

These results highlight the importance of balancing nutritional and structural properties in SSF systems. While SS provides the lipid-rich environment required for lipase induction, the addition of structural supports may improve the physical organization of the fermentation matrix. However, excessive dilution of the lipid fraction may reduce the induction effect and consequently decrease enzyme production. Therefore, the selection of substrate-support combinations in SSF should consider not only nutrient composition but also the physicochemical interactions governing microbial colonization and mass transfer within the solid matrix.

#### Effect of particle size and support addition

**Fig. 7:**
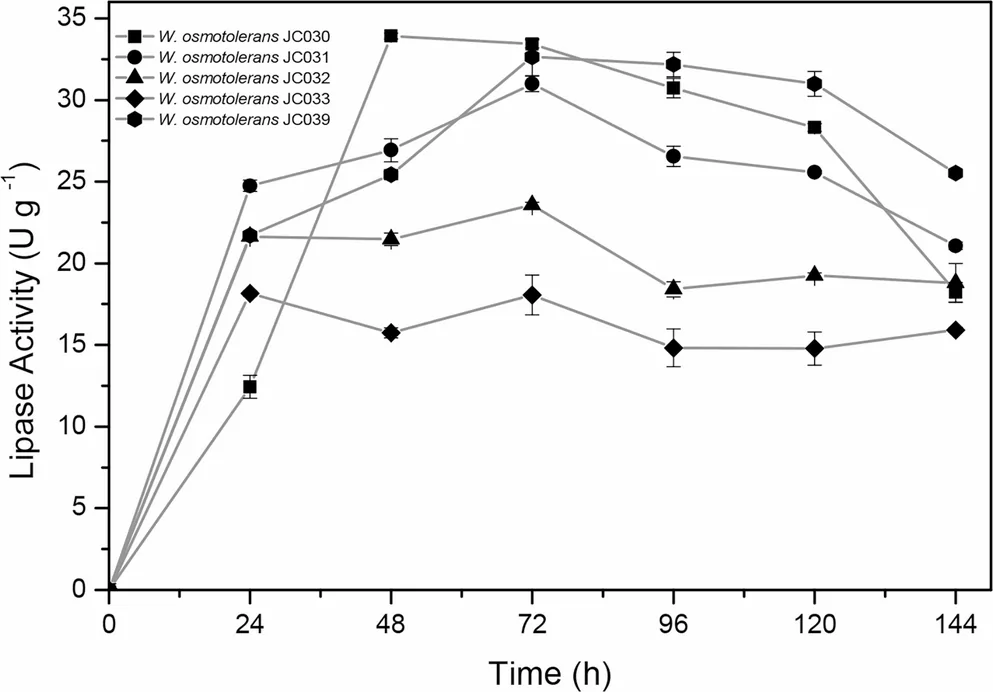
Lipolytic activity of five *Wickerhamiella osmotolerans* isolates during solid-state fermentation using sunflower seed with a particle size of 1.18–2.00 mm, 60% initial moisture, and incubation at 30 °C for 144 h. Values represent means, and error bars represent standard deviations from three independent fermentations (*n* = 3)

All five selected isolates produced lipase under SSF conditions using sunflower seed as the substrate, although their maximum activities and the times required to reach them differed (Fig. 7). JC030 exhibited the highest lipolytic activity, reaching 35.39 ± 0.43 U g⁻¹ at 48 h. JC039 reached 32.93 ± 1.94 U g⁻¹ at 72 h, followed by JC031 with 30.95 ± 0.83 U g⁻¹ at 72 h, JC032 with 23.57 ± 0.66 U g⁻¹ at 48 h, and JC033 with 18.16 ± 1.21 U g⁻¹ at 24 h. Thus, JC030 was selected for the subsequent SSF experiments because it combined the highest measured activity with an earlier production peak than JC031 and JC039. These results demonstrate isolate-level variability in lipase production under the same cultivation conditions, consistent with previous screening studies reporting substantial differences in lipolytic activity among microbial isolates [46].

The lipase production profile of *W. osmotolerans* JC030 showed a maximum activity at 48 h, followed by a gradual decrease during prolonged fermentation. This behavior is commonly observed in SSF processes and may be associated with nutrient depletion, accumulation of metabolic by-products, changes in substrate accessibility, or partial enzyme inactivation over time [46]. In addition, prolonged cultivation under low-moisture conditions may alter the physicochemical properties of the fermentation matrix, negatively affecting microbial activity and enzyme stability.

**Fig. 8:**
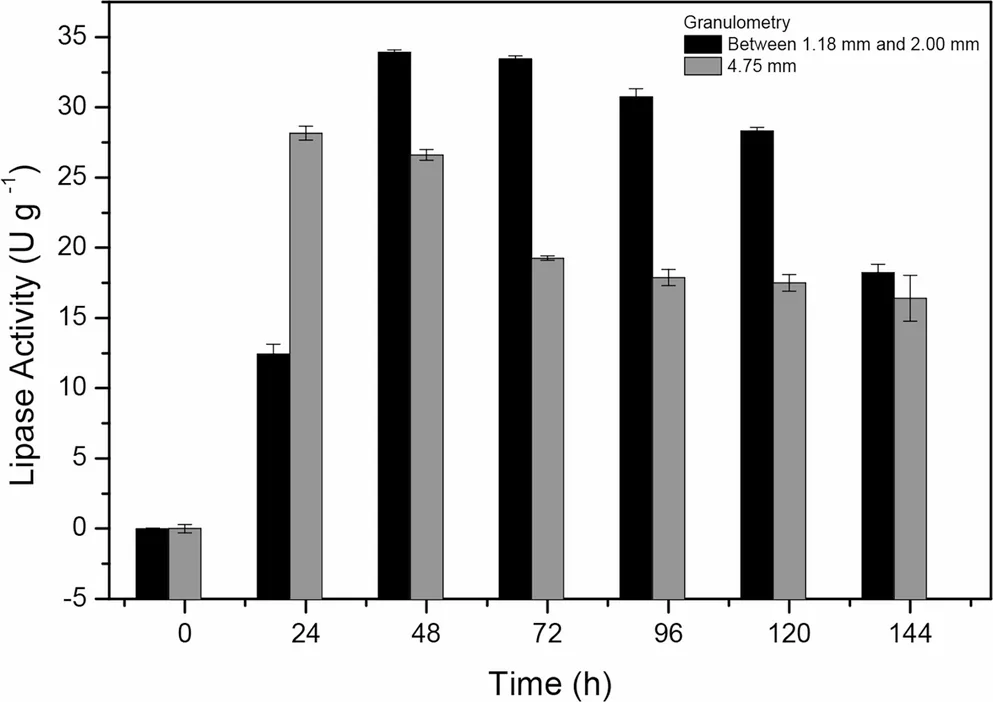
Lipolytic activity of *Wickerhamiella osmotolerans* JC030 during solid-state fermentation using sunflower seed with particle sizes of 1.18–2.00 mm and 4.75 mm. Fermentation was conducted at 60% initial moisture and 30 °C for 144 h. Values represent means, and error bars represent standard deviations from three independent fermentations (*n* = 3)

Particle size influenced both the magnitude and temporal profile of lipase production (Fig. 8). The 1.18–2.00 mm particle fraction reached the highest activity of 35.39 U g⁻¹ at 48 h. After this peak, activity decreased gradually but remained at 29.16 U g⁻¹ after 144 h. In contrast, the 4.75 mm particles reached their maximum activity earlier, attaining 25.88 U g⁻¹ at 24 h, followed by a more pronounced reduction during the subsequent fermentation period.

The 1.18–2.00 mm fraction produced a higher maximum activity, whereas the 4.75 mm particles resulted in an earlier production peak, demonstrating that particle size affected both the magnitude and kinetics of lipase production. Particle-size-dependent changes in SSF performance have also been reported in other fermentation systems [47]. Because surface area, porosity, aeration, and heat transfer were not measured in the present study, the mechanism underlying this effect cannot be determined. Based on its higher maximum activity and greater activity retention during fermentation, the 1.18–2.00 mm fraction was selected for the subsequent experiments.

**Fig. 9:**
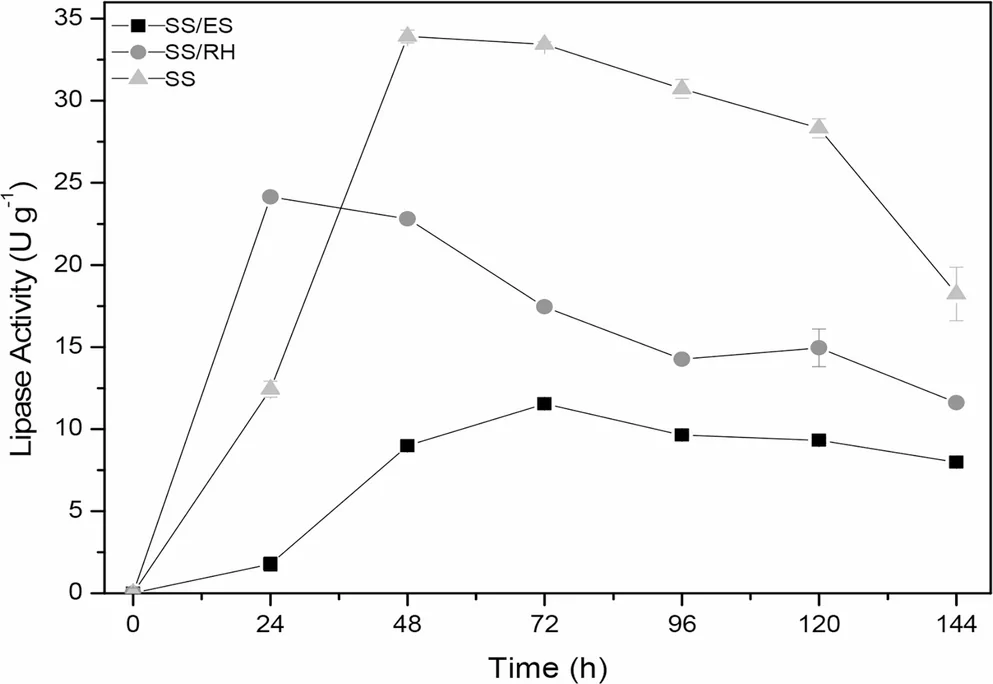
Lipolytic activity of *Wickerhamiella osmotolerans* JC030 during solid-state fermentation using sunflower seed alone (SS) or sunflower seed combined with rice husk (SS/RH, 1:1) or eggshell (SS/ES, 1:1). Sunflower seed particles ranged from 1.18 to 2.00 mm. Fermentation was conducted at 60% initial moisture on a wet basis and 30 °C for 144 h. Values represent means, and error bars represent standard deviations from three independent fermentations at each sampling time (*n* = 3)

The evaluation of substrate-support combinations further demonstrated the influence of matrix composition on lipase production (Fig. 9). The highest lipolytic activity was obtained with 100% sunflower seed, demonstrating that SS/RH was not the highest-performing condition in terms of enzyme production. Nevertheless, the SS/RH mixture reached 24.14 U g⁻¹ while replacing 50% of the sunflower seed with rice husk. Therefore, SS/RH was selected as a practical compromise between maintaining lipase production and increasing the proportion of agro-industrial residue in the fermentation matrix. This selection criterion does not demonstrate superior economic or environmental performance, which would require specific techno-economic and life-cycle assessments.

In contrast, the SS/ES system showed lower lipolytic activity (11.55 U g⁻¹). Its high ash and low oil contents indicate a markedly different matrix composition from SS/RH; however, the specific physicochemical mechanism responsible for the lower activity was not evaluated.

### Effects of SSF conditions on lipolytic activity

#### Effect of initial moisture content

Two-way ANOVA revealed significant effects of substrate composition (F₂,₁₈ = 117.81, *p* < 0.001), initial moisture content (F₂,₁₈ = 25.32, *p* < 0.001), and the substrate × moisture interaction (F₄,₁₈ = 9.41, *p* < 0.001) on lipolytic activity (Table 5). The significant interaction indicates that the effect of moisture depended on the substrate/support system.

**Fig. 10:**
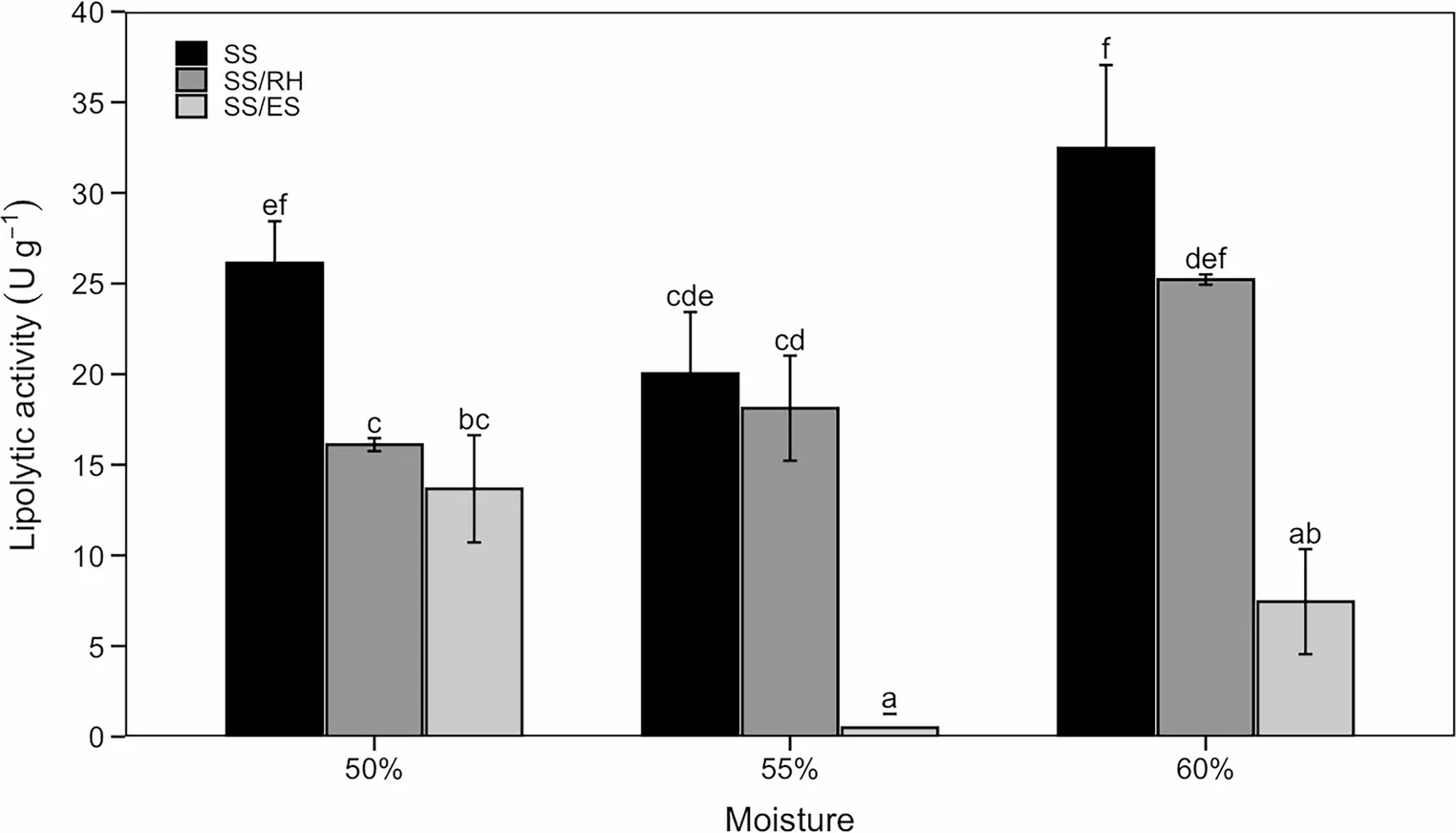
Effect of initial moisture content and substrate composition on lipolytic activity during SSF by *Wickerhamiella osmotolerans* JC030. Values represent mean ± standard deviation from three independent fermentations (n = 3). Different lowercase letters indicate significant differences among substrate–moisture combinations according to Tukey’s test (*p* < 0.05). SS, sunflower seed; SS/RH, sunflower seed/rice husk; SS/ES, sunflower seed/eggshell

The highest mean activity was obtained with SS at 60% initial moisture (32.47 ± 4.58 U g⁻¹). Within the SS/RH system, activity increased from 16.11 ± 0.36 U g⁻¹ at 50% moisture to 18.12 ± 2.90 U g⁻¹ at 55% and 25.22 ± 0.28 U g⁻¹ at 60%. The value obtained at 60% was significantly higher than that obtained at 50%, but did not differ significantly from that obtained at 55%. Therefore, 60% initial moisture was selected for the subsequent SS/RH experiments based on the highest observed mean rather than as a general optimum for all substrate systems (Fig. 10).

**Table 5:** Two-way ANOVA for the effects of substrate composition and initial moisture content on lipolytic activity

Source	df	Sum of squares	Mean square	F-value	*p*-value
Substrate	2	1682.53	841.27	117.81	< 0.001
Moisture	2	361.63	180.82	25.32	< 0.001
Substrate × moisture	4	268.64	67.16	9.41	< 0.001
Residual	18	128.53	7.14	—	—

The significant substrate × moisture interaction demonstrates that moisture should not be interpreted independently of substrate composition. Water availability affects substrate hydration, nutrient diffusion, matrix porosity, and oxygen transfer; consequently, the moisture condition associated with higher activity may differ among fermentation matrices [11, 48, 49].

#### Effect of substrate composition and inducer addition

A separate 2 × 3 factorial experiment was conducted to evaluate the effects of substrate composition and inducer condition on lipolytic activity. The experimental matrix comprised two substrate/support systems (SS/RH and SS/ES) and three inducer conditions (olive oil, sunflower oil, and no external inducer), with three independent fermentations for each combination. The 18 individual experimental results are presented in Table 6.

**Table 6:** Experimental matrix and lipolytic activity obtained for the substrate composition and inducer treatments

Experiment	Substrate (X1)	Inducer (X2)	LA (U g^− 1^)
1	SS/RH	OO	20.97
2	SS/RH	OO	21.33
3	SS/RH	OO	21.20
4	SS/RH	N/I	21.71
5	SS/RH	N/I	20.79
6	SS/RH	N/I	21.62
7	SS/RH	SO	19.03
8	SS/RH	SO	24.16
9	SS/RH	SO	18.49
10	SS/ES	OO	8.44
11	SS/ES	OO	8.30
12	SS/ES	OO	8.28
13	SS/ES	N/I	7.27
14	SS/ES	N/I	6.30
15	SS/ES	N/I	9.97
16	SS/ES	SO	5.42
17	SS/ES	SO	7.94
18	SS/ES	SO	6.28

**Fig. 11:**
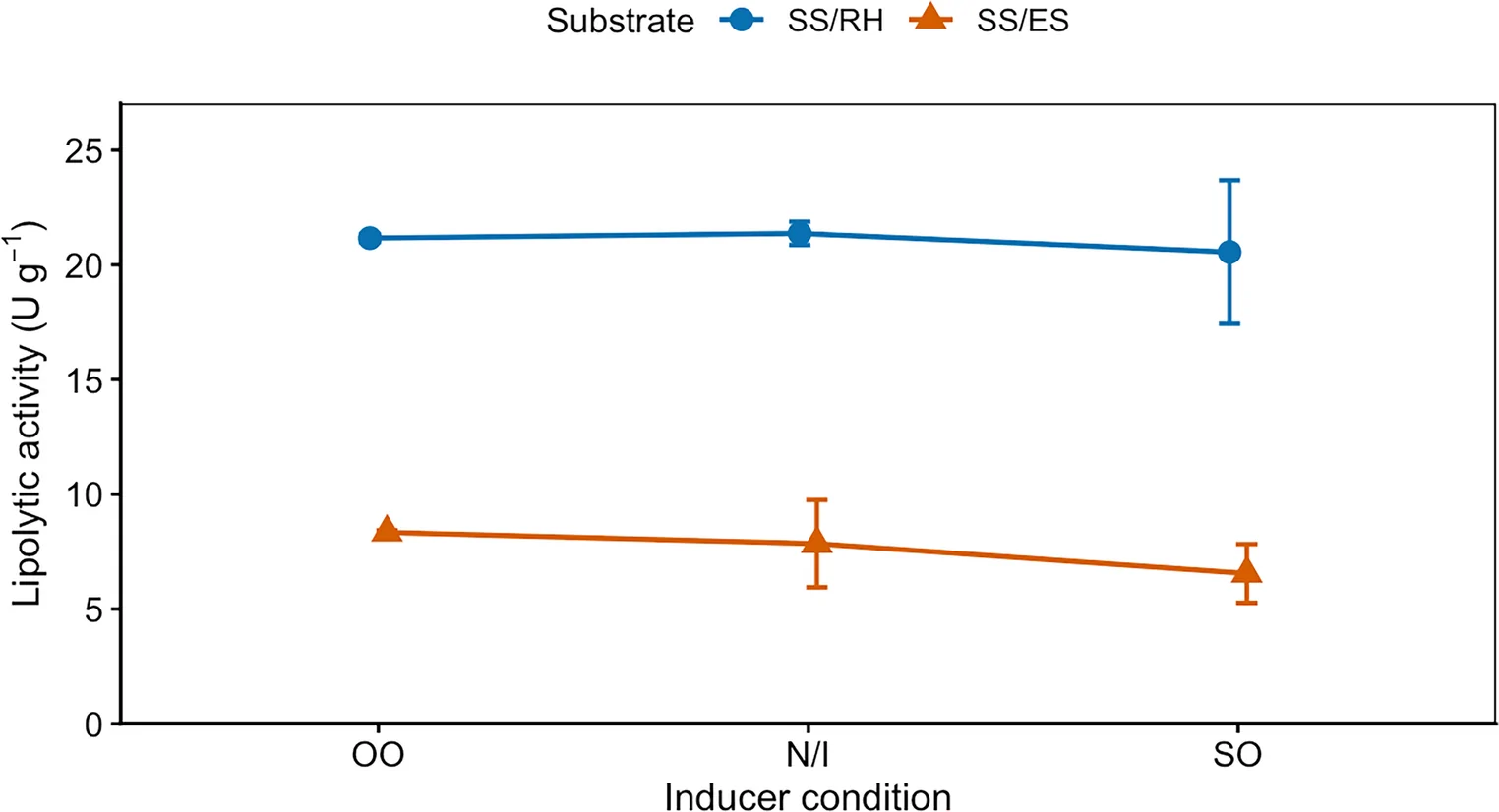
Effect of substrate composition and inducer condition on lipolytic activity. Values represent mean ± standard deviation of three independent fermentations. SS/RH, sunflower seed/rice husk; SS/ES, sunflower seed/eggshell; OO, olive oil; N/I, no inducer; SO, sunflower oil

The individual results obtained for each substrate–inducer combination are presented in Table 6. For SS/RH, the mean activities were 21.17 ± 0.18 U g⁻¹ with OO, 21.37 ± 0.51 U g⁻¹ without an external inducer, and 20.56 ± 3.13 U g⁻¹ with SO. For SS/ES, the corresponding activities were 8.34 ± 0.09, 7.85 ± 1.90, and 6.55 ± 1.28 U g⁻¹, respectively. Although the highest individual value was obtained with SS/RH and SO (24.16 U g⁻¹), inducer condition did not significantly affect mean lipolytic activity (Fig. 11).

Two-way ANOVA demonstrated a significant effect of substrate composition on lipolytic activity (F₁,₁₂ = 318.49, *p* < 0.001; Table 7). The overall mean activity obtained with SS/RH was 21.03 U g⁻¹, compared with 7.58 U g⁻¹ for SS/ES. In contrast, neither inducer condition (F₂,₁₂ = 1.01, *p* = 0.394) nor the substrate × inducer interaction (F₂,₁₂ = 0.21, *p* = 0.815) was significant. Therefore, no evidence was obtained that the addition of an external inducer improved lipolytic activity under the evaluated conditions.

**Table 7:** Two-way ANOVA for the effects of substrate composition and inducer condition on lipolytic activity

Source	df	Sum of squares	Mean square	F-value	*p*-value
Substrate	1	814.7339	814.7339	318.493	< 0.001
Inducer	2	5.1542	2.5771	1.007	0.394
Substrate × inducer	2	1.0675	0.5338	0.209	0.815
Residual	12	30.6971	2.5581	—	—

The consistently higher activity obtained with SS/RH than with SS/ES indicates that the support component influenced fermentation performance. Because both systems contained the same proportion of sunflower seed, the difference cannot be attributed solely to the availability of the lipid-rich substrate. Differences in matrix structure, aeration, water distribution, or nutrient accessibility may have contributed, although these mechanisms were not directly evaluated.

**Fig. 12:**
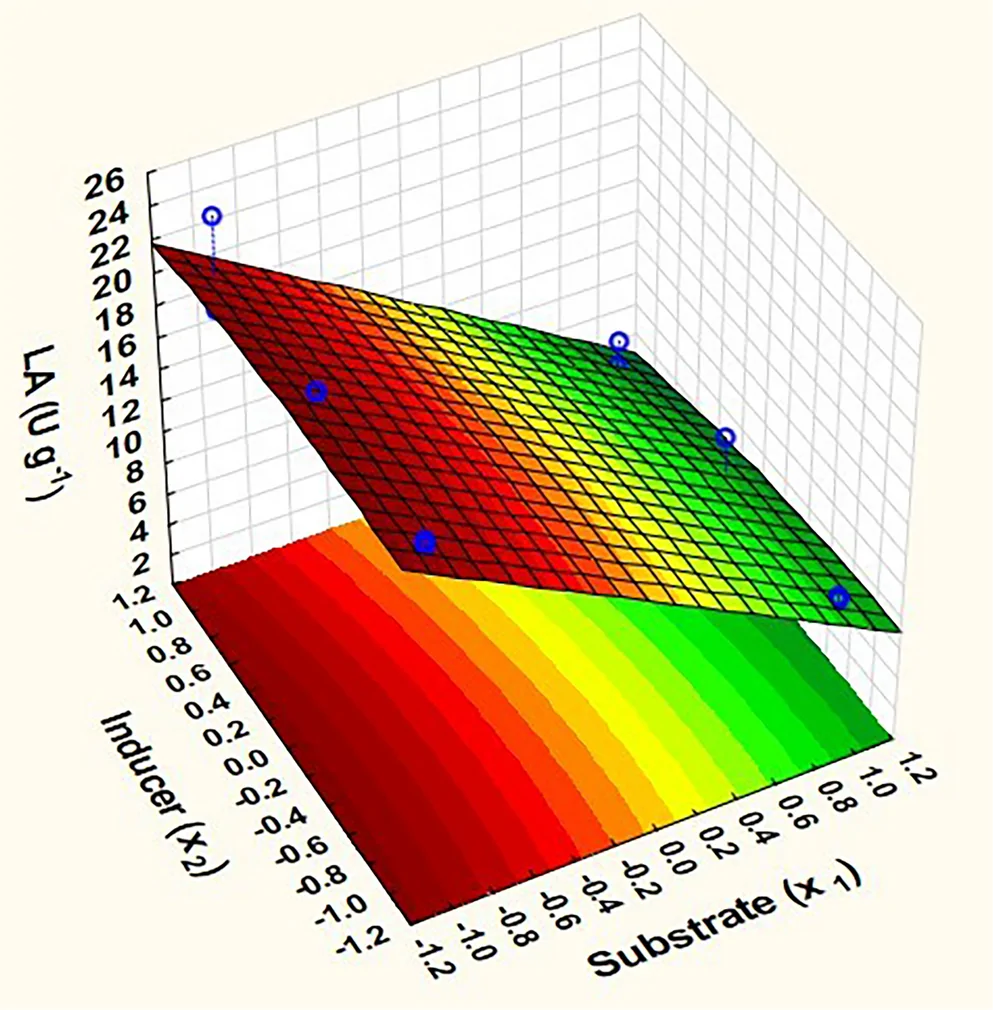
Response surface of the LA obtained from the mixed factorial experimental design with two- and three-level factors

The absence of significant inducer and substrate × inducer effects indicates that adding olive or SO did not improve mean lipolytic activity. Therefore, the no-inducer condition was selected because it provided activity comparable to that obtained with external inducers while simplifying the fermentation medium. The selected SS/RH condition was subsequently used to produce the fermented solids applied to the dairy wastewater (Fig. 12).

### Treatment with lipase-rich fermented solids followed by coagulation/flocculation/sedimentation

Dairy wastewater treatment was evaluated in two sequential stages. First, lipase-rich fermented solids produced by *W. osmotolerans* JC030 were applied at a dosage corresponding to 180 U L⁻¹ of lipolytic activity. The resulting fermented-solid-treated effluent (FSE) was subsequently subjected to C/F/S. Table 8 presents the physicochemical characteristics of the raw effluent (RE), FSE, and the effluent obtained after the subsequent C/F/S treatment, together with a stage-resolved accounting of changes in aqueous-phase O&G concentration and the corresponding apparent removal efficiencies.

Repeated-measures ANOVA showed a significant effect of treatment stage on pH (F₂,₄ = 18,417.00, *p* < 0.001), turbidity (F₂,₄ = 103,773.86, *p* < 0.001), O&G (F₂,₄ = 20,840.33, *p* < 0.001), and FFA (F₂,₄ = 54,273.00, *p* < 0.001). Holm-adjusted pairwise comparisons indicated differences among all three stages for pH, turbidity, and O&G. For FFA, RE differed from FSE and FSE + C/F/S, whereas FSE and FSE + C/F/S did not differ significantly.

**Table 8:** Stage-resolved physicochemical characterization and aqueous-phase O&G accounting during dairy wastewater treatment with lipase-rich fermented solids followed by C/F/S

Parameters	RE	FSE	FSE + C/F/S
pH	6.47 ± 0.04ᶜ	7.37 ± 0.05ᵃ	7.00 ± 0.04ᵇ
Turbidity (NTU)	1180 ± 15^b^	1190 ± 16ᵃ	693 ± 13^c^
O&G (mg L^− 1^)	79.2 ± 1.4ᵃ	27.4 ± 0.8ᶜ	31.2 ± 0.8ᵇ
FFA (% w/v as oleic acid)	0.64 ± 0.04ᵇ	1.98 ± 0.05ᵃ	1.99 ± 0.05ᵃ
O&G change relative to the previous stage (mg L⁻¹)	-	−51.8	+ 3.8
Net apparent O&G removal relative to RE (%)	-	65.4	60.6

Legend: Negative values for stage-wise O&G change indicate a decrease in the aqueous-phase concentration, whereas positive values indicate an increase relative to the preceding treatment stage. Apparent O&G removal was calculated as $\:\left[\left({C}_{RE}-{C}_{t}\right)/{C}_{RE}\right]\times\:100$, where $\:{C}_{RE}$ is the O&G concentration in the RE and $\:{C}_{t}$ is the concentration after the corresponding treatment.

Table 8 provides a stage-resolved accounting of the aqueous-phase O&G concentration. Treatment with the lipase-rich fermented solid decreased O&G from 79.2 to 27.4 mg L⁻¹, corresponding to an apparent removal of 65.4%. The subsequent C/F/S step increased the measured O&G concentration from 27.4 to 31.2 mg L⁻¹, representing a 13.9% increase relative to the FSE and reducing the net apparent removal to 60.6%. Therefore, C/F/S did not provide additional O&G removal under the evaluated conditions. Because the lipid fractions associated with the fermented solid, biomass, and settled material were not separately quantified, the cause of this increase cannot be determined from the available data.

The decrease in O&G during treatment with the lipase-rich fermented solid was accompanied by an increase in FFA from 0.64% to 1.98%, a pattern consistent with triglyceride hydrolysis. An increase in FFA concentration following the lipase-based treatment of dairy wastewater has also been reported by Golunski et al. [50]. After C/F/S, FFA remained essentially unchanged, whereas turbidity decreased from 1190 to 693 NTU, indicating that this subsequent stage primarily contributed to clarification. However, because an unfermented-solid control and a phase-resolved lipid mass balance were not included, the isolated contribution of lipase activity cannot be quantified. The possible introduction of FFA by the fermented solid and contributions from adsorption, physical retention, sedimentation, or biomass-associated effects cannot be excluded. Therefore, the results provide evidence consistent with lipid hydrolysis but do not demonstrate complete lipid degradation or mineralization.

The analytical parameters were selected to address the specific objectives of this proof-of-concept study. O&G and FFA were used to evaluate changes in the lipid fraction and provide evidence consistent with hydrolysis, whereas turbidity was used to assess the clarification achieved by C/F/S and pH was monitored as a relevant operational parameter. Together, these measurements allowed the stage-specific effects of the fermented-solid treatment and C/F/S to be distinguished. A broader characterization would be required for a comprehensive assessment of organic-load removal and regulatory compliance.

An additional limitation is that the wastewater-treatment experiments were conducted in three independent experimental units prepared from a single wastewater collection. Therefore, although the results demonstrate reproducibility within the evaluated batch, they do not account for the temporal and compositional variability commonly observed among different batches of industrial wastewater. Further studies using wastewater collected at different times are required to assess the robustness and broader applicability of the proposed treatment.

#### Effect on pH and turbidity

**Fig. 13:**
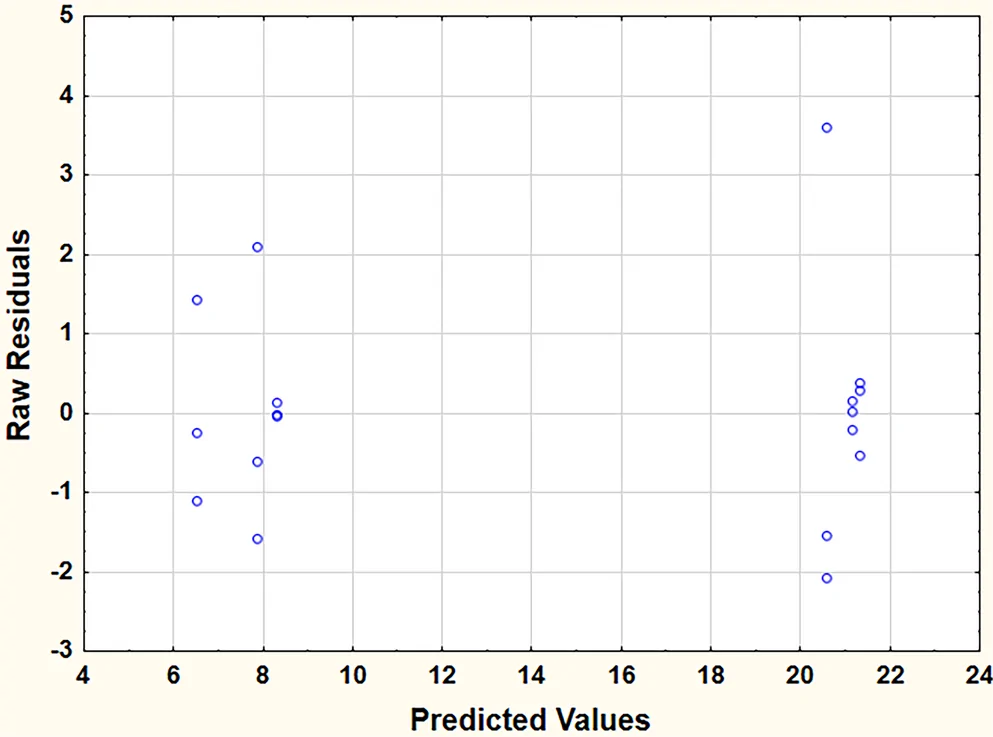
Residuals distribution vs predicted values from the model obtained in the mixed factorial experimental design with two and three level factors

Treatment with the lipase-rich fermented solid increased the pH from 6.47 to 7.37, followed by a decrease to 7.00 after C/F/S. This variation indicates that the treatment stages altered the acid–base equilibrium of the wastewater; however, the available data do not allow attribution to a specific mechanism. Treatment with the lipase-rich fermented solid did not reduce turbidity, which changed from 1180 to 1190 NTU. In contrast, the subsequent C/F/S step reduced turbidity to 693 NTU, corresponding to a stage-specific reduction of 41.8% and an overall reduction of 41.3% relative to the RE. Thus, the demonstrated contribution of C/F/S was clarification rather than additional O&G removal (Fig. 13).

### Comparison with previous enzymatic treatments and process implications

A comparable O&G reduction of 58.8% was reported by Bialeski et al. [22] for a dairy effluent treated with lipase produced by *Penicillium polonicum*. These comparisons provide contextual support for the treatment performance observed here but should be interpreted cautiously because the studies differed in wastewater composition, enzyme preparation, treatment conditions, and analytical procedures.

The direct application of the fermented solid avoided enzyme extraction and purification within the experimental workflow. A similar direct-use strategy has been reported for a dried solid enzymatic preparation applied to the hydrolysis of lipid-rich sewage scum [51]. This represents a potential operational simplification compared with processes based on recovered enzymes. However, reductions in processing cost, energy consumption, or environmental impact were not quantified and would require dedicated techno-economic and life-cycle assessments.

## Conclusion

This study identifies *Wickerhamiella osmotolerans* JC030 as a promising lipase-producing yeast for the generation of directly applicable fermented biocatalytic materials. Although 100% sunflower seed maximized lipolytic activity, the SS/RH system maintained relevant enzyme production while incorporating 50% rice husk and eliminating the need for an external lipid inducer. Direct application of the dried fermented solid resulted in 65.4% apparent aqueous-phase O&G removal, accompanied by FFA accumulation consistent with lipid hydrolysis. The subsequent C/F/S stage acted primarily as a clarification step, markedly reducing turbidity without providing additional O&G removal. These findings reveal distinct and complementary functions for the fermented-solid treatment and the physicochemical C/F/S stage and provide proof of concept for the extraction-free application of SSF-derived fermented solids in lipid-rich wastewater treatment. Further studies incorporating appropriate matrix controls, comprehensive wastewater characterization, and closed lipid mass balances are required to resolve the individual treatment mechanisms and assess process performance at larger scales.

## Data Availability

All data generated or analyzed during this study are included in this published article.

## Declarations

### Use of artificial intelligence

OpenAI’s image-generation tool, accessed through Codex, was used exclusively to create selected illustrative icons included in the graphical abstract. All photographic images were original materials provided by the authors. The authors assembled, edited, labeled, and verified the final graphical abstract and take full responsibility for its content. Artificial intelligence was not used to generate or modify experimental data, results, or scientific conclusions.
